# Enhancing detection of epileptic seizures using transfer learning and EEG brain activity signals

**DOI:** 10.1016/j.csbj.2025.10.054

**Published:** 2025-11-01

**Authors:** Erol Kına, Ali Raza, Prudhvi Chowdary Are, Carmen Lili Rodriguez Velasco, Julien Brito Ballester, Isabel de la Torre Diez, Naveed Anwer Butt, Imran Ashraf

**Affiliations:** a Van Yüzüncü Yıl University, Özalp Vocational School, Van, 65100, Türkey; b Department of Software Engineering, University of Lahore, 54000 Lahore, Pakistan; c BITSolutionsus, 665 Villa Creek, Dr Suite A218, Dallas, TX 75234, United States; d Universidad Europea del Atlantico, Isabel Torres 21, Santander, 39011, Spain; e Universidad Internacional Iberoamericana, Campeche, 24560, Mexico; f Universidad Internacional Iberoamericana, Arecibo, 00613, Puerto Rico; g Universidade Internacional do Cuanza, Cuito, Angola; h Universidad de La Romana, La Romana, Republica Dominicana; i Department of Signal Theory and Communications and Telematic Engineering, University of Valladolid, Paseo de Belen, 15, Valladolid, 47011, Spain; j Department of Computer Science, Faculty of Computing and Information Technology, University of Gujrat, Gujrat, Pakistan; k Department of Information and Communication Engineering, Yeungnam University, Gyeongsan, South Korea

**Keywords:** EEG signals, Brain activity, Epileptic seizures, Transfer learning, Explainable AI

## Abstract

Epileptic seizures are neurological events characterized by sudden and excessive electrical discharges in the brain, leading to disruptions in brain function. Epileptic seizures can lead to life-threatening situations such as status epilepticus, which is characterized by prolonged or recurrent seizures and may lead to respiratory distress, aspiration pneumonia, and cardiac arrhythmias. Therefore, there is a need for an automated approach that can efficiently diagnose epileptic seizures at an early stage. The primary objective of this study is to develop a highly accurate approach for the early diagnosis of epileptic seizures. We use electroencephalography (EEG) signal data based on different brain activities to conduct experiments for epileptic seizure detection. For this purpose, a novel transfer learning technique called random forest-gated recurrent unit (RFGR) is proposed. The EEG brain activity signal data is fed into the RFGR model to generate a new feature set. The newly generated features are based on the class prediction probabilities extracted by the RFGR and are utilized to train models. Extensive experiments are carried out to investigate the performance of the proposed approach. Results demonstrate that the RFGR, when used with the random forest model, outperforms state-of-the-art techniques, achieving a high accuracy of 99.00 %. Additionally, explainable artificial intelligence analysis is utilized to provide transparent and understandable explanations of the decision-making processes of the proposed approach.

## Graphical Abstract

**Figure:**
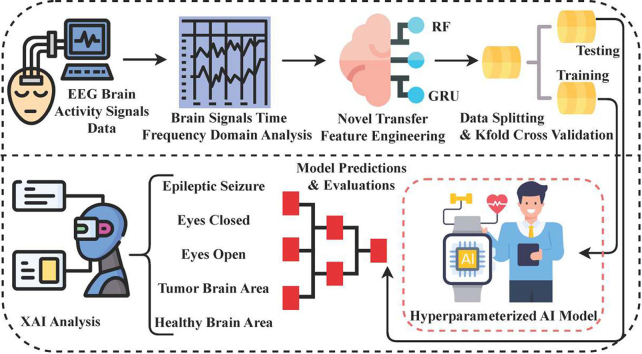

## 1 Introduction

Epileptic seizures are sudden electrical disturbances in the brain, signaled by various behavioral and physiological manifestations [1]. Epilepsy, a neurological disorder, is distinguished by recurrent seizures. During a seizure, there is a temporary disturbance in the brain’s normal functioning, leading to abnormal sensations, behaviors, or loss of consciousness. The precise cause of epilepsy is frequently undetermined, but it can be initiated by numerous factors, including genetic influences, brain trauma, infections, or developmental conditions. Seizures can vary in intensity and duration, ranging from mild and barely noticeable to severe and debilitating [2]. They can affect different brain areas, giving rise to diverse symptoms such as convulsions, muscle spasms, loss of awareness, changes in sensory perception, or unusual emotions. Understanding the nature of epileptic seizures is important for accurate diagnosis, treatment planning, and improving an individual’s quality of life with epilepsy. Ongoing research focuses on unraveling the underlying mechanisms, identifying potential risk factors, and developing innovative therapeutic approaches to manage and mitigate the impact of epileptic seizures [3].

Diagnosing epileptic seizures using electroencephalography (EEG) brain activity signals is an important and widely utilized method in clinical practice [4]. EEG is obtained by placing electrodes on the scalp to detect the brain’s electrical signals in a non-invasive manner. Epileptic seizures are marked by abnormal electrical activity in the brain, and EEG offers valuable insights into the patterns of these discharges over time and space [5]. By analyzing the EEG signals, clinicians can detect specific patterns associated with epileptic seizures, such as spike-and-wave complexes or sharp waves. Advanced signal processing techniques such as spectral analysis, time–frequency analysis, and machine learning (ML) approaches have been employed to extract features from EEG data signals and improve the performance accuracy of seizure detection [6]. Detection of epileptic seizures is important as studies have reported mortality rates ranging from 1.6 to 7.8 per 1000 person-years among individuals with epilepsy.

Artificial intelligence (AI) techniques have emerged as promising tools for diagnosing epileptic seizures using EEG brain activity signals [7]. EEG signals offer significant insights into the brain’s electrical activity and assist in detecting patterns linked to epileptic seizures. AI algorithms can extract meaningful information from complex, high-dimensional data such as EEG signals. Transfer learning [8], a technique where knowledge gained from one task is transferred to another related task, has proven particularly advantageous in EEG-based seizure detection. By leveraging the knowledge from other related tasks, transfer learning can effectively capture important patterns from EEG signals, enhancing the diagnostic accuracy of AI models. Advanced AI approaches for epileptic seizure detection using EEG brain activity signals hold immense potential. It enables healthcare professionals to leverage advanced methods to assist in detecting, classifying, and predicting seizures, ultimately helping to improve patient care [9].

### 1.1 Research objectives

The primary contributions of this research study for detecting epileptic seizures are as follows.

• This study proposes an automated approach utilizing EEG brain activity signals and ML approaches for the effective diagnosis of epileptic seizures. For this purpose, a novel transfer technique called random forest-gated recurrent unit (RFGR) is proposed for feature engineering. The EEG brain activity signals data are fed into the proposed RFGR model, which generates a feature set.
• The performance of the proposed RFGR approach is analyzed for epileptic seizure detection in comparison to other ML approaches. In addition, principal component analysis (PCA)-based features are also utilized with these models. To obtain optimal performance, the hyperparameters of each model are carefully tuned and validated using k-fold validation.
• Performance is analyzed against the existing state-of-the-art models. Additionally, the computational time of each technique is assessed to ensure computational efficiency. Experimental work infers that the proposed method achieves superior performance.
• To provide transparent and understandable explanations of the EEG decision-making processes by the proposed techniques, an explainable artificial intelligence (XAI) analysis is also carried out. This analysis utilizes the Shapley additive explanation (SHAP) chart and the model decision tree as the basis for providing insightful explanations.

The remaining sections of this paper are structured as follows: Section 2 offers a comparative review of pertinent literature and identifies gaps in current research. Section 3 delineates the materials and methods utilized in this investigation. Section 4 presents a comparative analysis of the study’s findings. Lastly, Section 5 elaborates on the final remarks and outcomes derived from this research.

## 2 Literature analysis

This section examines existing studies to provide insights into various methods and techniques used for epileptic seizure detection. The results of these studies, along with their pros and cons, are discussed here.

The study [10] focused on improving the precision of epileptic seizure disease detection by leveraging EEG signals and machine learning classifiers. The proposed approach involved utilizing a framework that employed a genetic algorithm with 54-DWT (discrete wavelet transform) mother wavelet feature analysis to examine the EEG signal data. The experimental findings demonstrate that the artificial neural network (ANN) classifier yields the highest accuracy of 97.00 % in identifying epilepsy seizures compared to the other classifiers, showcasing superior performance. Similarly, [11] proposed a novel approach to detect epilepsy seizures in real time using EEG data. The study employs DWT and PCA algorithms to extract relevant features from different frequency bands. The authors employ the RUSBoosted tree-based ensemble method to enable real-time seizure detection. The results demonstrate that the algorithm achieves 97.00 % accuracy using the UB three-class classification dataset.

In the study [12], authors explore the application of power data spectrum density analysis in resting-state values of EEGs to detect abnormalities in the brains of individuals diagnosed with psychogenic non-epileptic seizures (PNES). The study comprises a group of 20 PNES patients and 19 healthy subjects. The researchers employed three classification models to examine the functional connectivity features of the dataset. Among these models, the multilayer perceptron (MLP) demonstrated the maximum performance, achieving an accuracy of 91.02 %. Similarly, in [13], the authors used five distinct deep learning (DL) models to forecast epileptic seizures by leveraging intracranial EEG datasets. The applied models include convolutional neural networks (CNN), the fusion of two-CNNs, three-CNNs, four-CNNs, and transfer learning using ResNet50. Through experimentation, it was observed that the 3-CNN and 4-CNN models exhibit good performance results in contrast to the other models, achieving an accuracy of 95.00 %.

The study [14] presents an innovative wearable system that utilizes EEG, electrocardiogram (ECG), and photoplethysmogram (PPG) signals to predict epileptic seizures. The system underwent testing on patients with epilepsy in a clinical environment and employs ML models to categorize the individual’s condition as pre-seizure, normal, or seizure. By developing a simplified model based on boosted trees, the authors achieved a prediction accuracy of 91.50 %. A different investigation [15] presents an innovative method for epilepsy classification, employing ensemble ML techniques alongside a fuzzy logic inference system. This research utilizes health parameters derived from wearable-sensor data [16] to forecast occurrences of epilepsy. The experimental findings highlight the superior performance of the ensemble boosting method, in conjunction with the fuzzy data inference system, achieving an accuracy of 97.00 %.

The study [17] introduced a unique approach to epileptic disease seizure prediction using EEG data by combining a dense CNN (DenseNet) and long short-term memory (LSTM) in a hybrid DL approach. To prepare the data for input, the EEG signals are transformed into the time–frequency data domain using DWT. Subsequently, experiments are performed on the scalp EEG dataset named CHB-MIT. The evaluation results indicate that the proposed method achieves a prediction accuracy of 93.28 %. The authors in [1] present DL models capable of forecasting epileptic seizures by analyzing scalp EEG signals. These models utilize distinct learning approaches and are specifically designed to comprehensively understand the data obtained from multiple subjects comprehensively. The proposed Siamese model achieves an accuracy of 91.54 % when tested on the EEG dataset named CHB-MIT.

Along the same direction, a subject-independent seizure predictor is introduced in [18] that utilizes the geometric DL model. The predictor operates in two stages: first, by utilizing graph data derived from the physical connections within the EEG data grid, and subsequently, by generating subject-specific graph data using DL models. The proposed models achieve an accuracy of 95.38 % in predicting seizures with a one-hour advance notice. The findings were derived from assessing the models using the CHB-MIT-EEG benchmark dataset, incorporating data from 23 participants. Additionally, another investigation [19] explored the utilization of ML techniques with the UCI Epileptic Seizure dataset. The results reveal that the support vector machine (SVM) model outperformed other algorithms with hyperparameter optimization, exhibiting an accuracy rate of 97.86 %.

Research work [20] introduces a novel approach for epileptic seizure detection using a two-layer LSTM deep neural network. The model incorporates the Swish activation function and effectively extracts feature data from the time and frequency domains. Performance evaluation of the proposed model is conducted using the Melbourne dataset, demonstrating good outcomes. The model achieves a high area under the curve (AUC) score of 0.92 and exhibits a sensitivity and accuracy of 86 % for seizure detection. Another research [21] implemented two distinct DL models to detect seizures tailored to individual patients, utilizing CHB-MIT data. The initial model employed a one-dimensional CNN, while the second integrated a combination of LSTM and CNN networks. The results show that the proposed approach achieved a prediction accuracy of 94.83 %. Similarly, [22] applied the XGBoost approach for EEG-based seizure disease detection. The proposed method scores an accuracy of 86 % specifically with the CHB-MIT dataset.

Through the comparative analysis of the existing literature, we observed several research gaps related to epileptic seizure detection using EEG brain activity signal data. In particular, we identified the following research gaps.

• In the existing research, for the most part, traditional ML approaches are used for epileptic seizure detection. Also, simple signal data processing algorithms are used. There is a need to apply advanced transfer learning-based approaches.
• The performance of epileptic seizure detection is low in the analyzed literature. We identify that recent state-of-the-art methods achieve the highest accuracy of 97 %. Still, there is a need for performance enhancement in critical epileptic seizure detection.

We also perform a comparative analysis of the literature on epileptic seizure disease detection using EEG data, as demonstrated in Table 1. It highlights the approaches and datasets used in existing literature, as well as the reported performance. Considering the above-mentioned limitations, this study aims to build a model based on transfer learning to enhance the performance of epileptic seizure detection.

**Table 1:** Comparative analysis of discussed literature.

Ref.	Year	Dataset	Approach	Accuracy
[1]	2021	CHB-MIT-EEG dataset	Siamese model	91.54 %
[10]	2020	EEG signal data	ANN	97.00 %
[11]	2022	EEG data named UB	RUSBoosted tree ensemble	97.00 %
[12]	2021	Rest-EEG dataset	MLP classifier	91.02 %
[13]	2022	Intracranial EEG dataset	CNN	95.00 %
[14]	2022	Ear EEG, ECG and PPG signals	Boosted Trees	91.50 %
[15]	2022	Wearable sensor health record	Ensemble boosting classifier	97.00 %
[17]	2021	CHB-MIT scalp EEG dataset	hybrid Densenet and LSTM	93.28 %
[18]	2022	CHB-MIT-EEG dataset	Geometric deep learning	95.38 %
[19]	2021	UCI epileptic seizure dataset	SVM	97.86 %
[20]	2021	Melbourne dataset	Two-Layer LSTM	86.00 %
[21]	2023	CHB-MIT-EEG dataset	CNN and LSTM	94.83 %
[22]	2023	CHB-MIT dataset	XGBoost	86.00 %

The proposed research methodology is evaluated using the XAI approach [23]. XAI plays an important role in enhancing the diagnosis of epileptic seizures. Traditional diagnostic methods often rely on manual interpretation of EEG recordings, which can be time-consuming and subjective. However, XAI algorithms have emerged as a promising approach to assist clinicians in accurately and efficiently diagnosing epileptic seizures. XAI algorithms provide clinicians with interpretable insights into the decision-making process, enabling them to understand the reasons behind automated diagnoses and predictions. This interpretability is crucial in epilepsy diagnosis as it allows clinicians to validate the model’s predictions and gain confidence in its reliability. XAI models can highlight specific regions or features in EEG signals that contribute to the prediction of epileptic seizures.

## 3 Proposed methodology

**Fig. 1:**
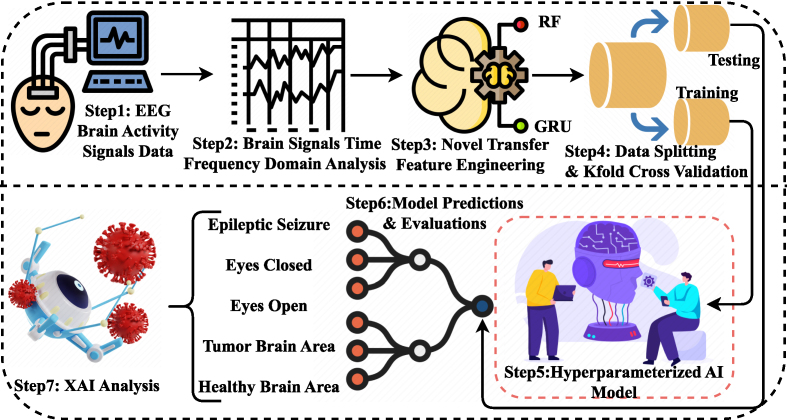
The workflow of the proposed epileptic seizure disease detection methodology.

In this section, we present the proposed research methodology. We provide a descriptive analysis of the materials and methods employed for the diagnosis of epileptic seizure disease. The workflow of the proposed methodology is illustrated in Fig. 1. The stepwise description of the methodology is provided in the subsequent sections.

The proposed approach executes the following steps.

• **Step-1:** EEG recordings from subjects with epileptic seizures will be considered for experiments. The dataset is based on brain activity classes, including ’eyes closed’, ’epileptic seizure’, ’eyes open’, ’tumor brain area’, and ’healthy brain area’.
• **Step-2:** Brain signal analysis in the time–frequency domain is applied to examine and interpret brain signals in both the temporal and spectral domains. By analyzing brain signals in the time–frequency domain, we can gain important insights and data into the dynamic changes and interactions occurring within the brain.
• **Step-3:** A novel transfer technique called RFGR is proposed for feature engineering. The signal data is fed into the proposed model, which generates a new feature set for epileptic seizure detection.
• **Step-4:** The transfer feature set is first split into training (80 %) and testing (20 %) subsets to apply the techniques. The feature extraction stage (RFGR) was applied only within the training data during each fold, and the corresponding transformation was then applied to the respective test split. The class posterior probabilities used in RFGR were always computed using training samples only, ensuring that no information from the test data was used during feature construction. Additionally, k-fold cross-validation is employed to ensure the robustness and generalizability of the methods used.
• **Step-5:** The hyperparameters fine-tuning is applied for learning approaches to obtain optimal performance.
• **Step-6:** The models are used for epileptic seizure detection on unseen testing data. The proposed approach is evaluated using several performance evaluation parameters.
• **Step-7:** The analysis of the results with the proposed model is conducted using explainable AI to comprehend the decision-making mechanisms of the model.

### 3.1 Rationale of the proposed approach

This study employed transfer learning combined with EEG brain activity signals because this approach effectively addresses two major challenges in epileptic seizure detection:

i The scarcity of large, labeled EEG datasets, and
ii The high variability in EEG patterns across individuals. Traditional machine learning models often require extensive subject-specific training data, which is not always feasible in clinical practice.

By applying transfer learning, we leveraged knowledge from pre-trained deep neural networks to extract robust and generalizable features from EEG signals. This allowed us to significantly reduce the dependency on large subject-specific datasets while still achieving high detection accuracy. Moreover, transfer learning enabled us to adapt existing models to the unique frequency and temporal characteristics of EEG data, thus improving model performance on seizure detection tasks.

We also selected this method because EEG data is inherently complex and non-linear. Deep learning models enhanced through transfer learning have been shown to capture subtle temporal-spatial dependencies more effectively than traditional statistical methods. This aligns with recent advancements in biomedical signal processing, where transfer learning has emerged as a powerful technique for improving diagnostic tools.

### 3.2 EEG brain activity signals data

The EEG brain activity signals benchmark data [24] is utilized to evaluate the proposed model. The initial dataset consisted of five folders, each containing 100 files that represented individual subjects. Each file documented brain activity data over a period of 23.6 s. These recorded data were sampled into the 4,097 data points, each point corresponding to a specific moment in the EEG recording.

Consequently, the dataset included 500 subjects, each with 4097 data points over 23.5 s. For ease of analysis, the dataset was divided into 23 chunks, each containing data of 178 points, which is equal to 1 s of EEG recording. These chunks were shuffled to ensure randomization, resulting in a dataset with 11,500 observations. Each observation consists of 178 data features representing 1 s of EEG recording, with the final column representing the label (y). The columns from X1 to X178 represent the explanatory variables within the dataset. The sampling rate of the EEG dataset is 178 Hz. The dataset consists of one channel, representing a single EEG signal recording per subject. The CSV format dataset is used for conducting this research experiment.

Concerning the labeling of the dataset, the seizure events were annotated by qualified neurologists. Therefore, the ground-truth labels provided in this dataset are based on expert medical annotations, ensuring the reliability of the data used for experiments.

The distribution analysis of the target classes reveals that all brain activity classes, including ‘eyes closed’, ‘epileptic seizure’, ‘eyes open’, ‘tumor brain area’, and ‘healthy brain area’, have a sample size of 2,300 in the dataset. It is important to point out that all subjects falling into classes such as eyes closed, eyes open, tumor brain area, and healthy brain area did not experience epileptic seizures. The analysis concludes that all target classes have equal data samples, indicating a balanced distribution of records. Concerning ’tumor brain area’ and ’healthy brain area’, the following information is important.

• Tumor Brain Area: This refers to EEG signals recorded from regions of the brain affected by a tumor. These signals help in understanding the neural activity associated with abnormal brain tissue due to tumor presence.
• Healthy Brain Area: This indicates EEG signals collected from regions of the brain that are unaffected by any pathological conditions, representing normal brain activity.

In the context of the dataset, these categories help differentiate between pathological and non-pathological brain regions, allowing us to analyze and classify brain activity patterns more effectively.

### 3.3 Brain signals analysis in time-frequency domain

**Fig. 2:**
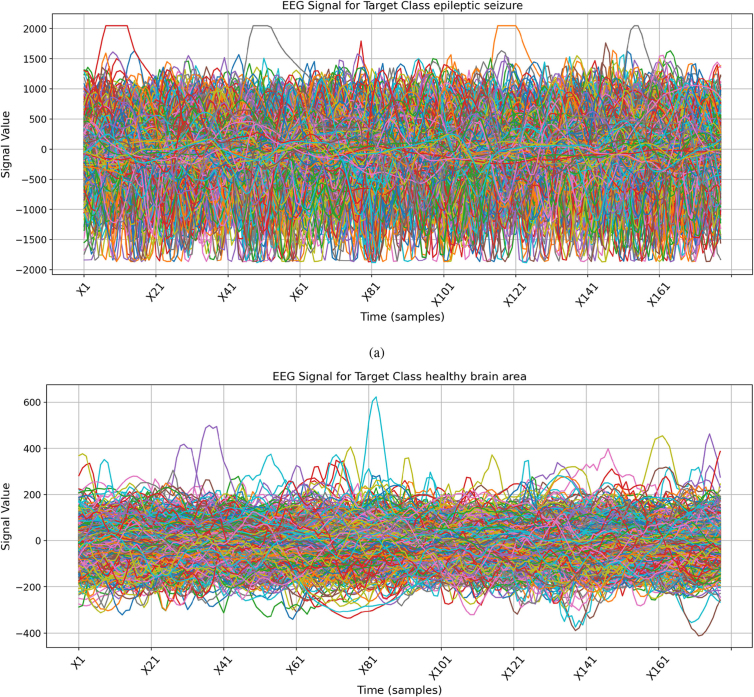
The EEG brain signals analysis in the time-frequency domain for different classes, **(a)** Epileptic seizure, **(b)** Healthy area.

Brain signal analysis in the time–frequency domain involves examining and interpreting brain signals in both the temporal and spectral domains. We can gain valuable insights into the dynamic changes and interactions occurring within the brain by analyzing brain signals in the time–frequency domain, as illustrated in Fig. 2.

The analysis illustrated that the epileptic seizure class has the maximum and minimum EEG brain activity signal values, ranging between 2000 and −2000. The same applies to the tumor brain area class in the time–frequency domain. In the tumor brain area class, most signal occurrences lie between the EEG brain activity signal values of 500 and −500. Only some of the signals deviate from this pattern, indicating affected brain regions. The remaining brain activity signals, EEG values, exhibit patterns ranging from 300 to −400. This analysis indicates that all brain activity signals have patterns of oscillatory activity that indicate various cognitive processes.

### 3.4 Novel transfer feature fusion approach

**Fig. 3:**
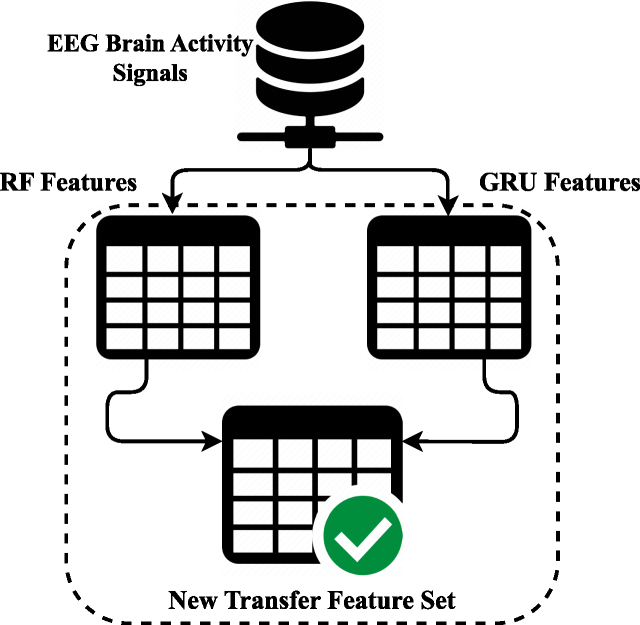
Novel proposed feature generation mechanism analysis.

The proposed transfer feature fusion approach is examined in this section. Fig. 3 illustrates the proposed RFGR approach. The original feature data of EEG brain activity signals are input in parallel to an ML model and a DL neural network for transfer feature extraction. First, the RF class prediction probabilities are used to create a feature set by inputting EEG brain activity signals. Then, temporal features are extracted using the gated recurrent unit (GRU) by inputting EEG brain activity signals. Finally, a new hybrid transfer feature set is formed by combining the RF and GRU methods’ output features. This study uses the newly created transfer features to build the applied neural network approaches.

To begin with, the RF method for class prediction probabilities, let’s consider a dataset with N samples and M features. Each sample data is represented by a feature vector $x_{i}=(x_{i1},x_{i2},\ldots ,x_{iM})$, where 1≤i≤N.

An RF consists of T decision trees. Each decision tree points are constructed by selecting a random subset of training samples and a random subset of features for each split. The goal is to learn a mapping from feature vectors to class probabilities. For a given sample $x_{i}$, the RF predicts the class probabilities $\hat{P}(y_{j}|x_{i})$ for each class $y_{j}$, where 1≤j≤K (assuming K classes). The RF predicts class probabilities $\hat{P}(y_{j}|x_{i})$ for each class $y_{j}$, where 1≤j≤K. The accumulated probabilities are normalized to produce the final prediction:

(1) $$\hat{P}(y_{j}|x_{i})=\frac{1}{T}\sum_{t=1}^{T}P_{t}(y_{j}|x_{i})$$

where $P_{t}(y_{j}|x_{i})$ is the predicted probability from the tth decision tree.

To compute the class prediction probabilities, the RF employs the following steps:

i For each decision tree t value in the forest:
  (a) Randomly select a subset of n training samples $D_{t}$ from the original dataset.
  (b) Train the decision tree t using the samples in $D_{t}$.
ii For each sample $x_{i}$:
  (a) Initialize the class probability estimates $\hat{P}(y_{j}|x_{i})$ for each class $y_{j}$ to zero.
  (b) For each decision tree sample t value in the forest:
    i. Traverse the decision tree t to determine the leaf node $l_{t}$ into which $x_{i}$ falls into.
    ii. Accumulate the class probability estimates from the leaf node $l_{t}$ to $\hat{P}(y_{j}|x_{i})$ for each class $y_{j}$.
  (c) Normalize the accumulated class probability estimates $\hat{P}(y_{j}|x_{i})$ to obtain the final class prediction probabilities.

The RF model provides a robust and effective approach for extracting class prediction probabilities as features from input data. In addition, the GRU model is used for temporal feature extraction from the EEG brain activity signals data. The following are mathematical notations for GRU that denote the temporal feature extraction from signal data.

The GRU processes the EEG signal data to extract temporal features. Given the input $x_{t}$ at time t and the hidden state $h_{t-1}$, the following equations describe the GRU’s operations:

(2) $$z_{t}=\sigma (W_{z}x_{t}+U_{z}h_{t-1}+b_{z})$$

(3) $$r_{t}=\sigma (W_{r}x_{t}+U_{r}h_{t-1}+b_{r})$$

(4) $$h_{t}=(1-z_{t})⊙h_{t-1}+z_{t}⊙\tanh(W_{h}x_{t}+U_{h}(r_{t}⊙h_{t-1})+b_{h})$$

Where:

• $z_{t}$ and $r_{t}$ are the update and reset gates, respectively.
• σ denotes the sigmoid activation function.
• ⊙ represents element-wise multiplication.
• W, U, and b are learnable parameters.

Algorithm 1 expresses the step-by-step working of the proposed transfer learning approach. The proposed approach receives EEG brain activity signals as input and generates a new transfer feature set.

Algorithm 1 **RFGR algorithm**

The novelty of the RFGR approach lies in its hybrid transfer feature extraction technique, which combines the strengths of both RF and GRU models to enhance the diagnostic performance in detecting epileptic seizures. Specifically, it offers the following advantages.

• **Dual Feature Extraction**: Unlike conventional methods that rely solely on either ML or DL techniques, the RFGR model leverages both approaches simultaneously. The RF model captures nonlinear patterns and feature importance, while the GRU model efficiently extracts temporal dependencies from sequential EEG data.
• **Transfer Feature Integration**: The prediction probabilities from the RF model are utilized as new informative features alongside the temporal features extracted from the GRU model. This integration creates a richer, hybrid feature set that effectively combines interpretable machine learning features with complex temporal dynamics learned by the GRU.

### 3.5 Applied artificial intelligence techniques

In this research, we used many DL and ML models to diagnose epileptic seizures and other brain activity efficiently. The applied ML models are support vector machine (SVM), logistic regression (LR), Gaussian Naive Bayes (GNB), and RF [25], [26], [27]. The GRU and LSTM are applied as DL models for comparison. The architecture of DL models is shown in Table 2.

**Table 2:** The GRU and LSTM models architectures.

Layer type	GRU model		LSTM model	
	Output Shape	Params	Output Shape	Params
GRU	(None, 64)	12,864	–	–
LSTM	–	–	(None, 64)	16,896
Dropout	(None, 64)	0	(None, 64)	0
Dense	(None, 5)	325	(None, 5)	325
**Total Params**	–	**13,189**	–	**17,221**

### 3.6 Explainable artificial intelligence

In this research, the Shapley values and TreeMap approaches [28] are utilized to interpret the estimation decisions of the proposed model. The SHAP [29] is an approach used to understand the contributions of each data feature in determining the predictions made by the proposed model and produces graphical results. In the TreeMap approach, the dataset is partitioned into rectangular regions based on feature space, creating a hierarchical structure of nested rectangles. Each rectangle represents a region in the feature space, and the classification task is performed by assigning a label to each region. This approach provides an intuitive visualization of the decision boundaries.

### 3.7 Hyperparameter settings

The hyperparameter settings of the models for EEG-based seizure detection are crucial for achieving accurate and reliable results [30]. Research findings indicate that fine-tuning hyperparameters can significantly enhance the accuracy and predictive capabilities of EEG-based seizure detection models. The hyperparameters of the used models are given in Table 3. The selected hyperparameters were optimized using a randomized search approach, combined with a recursive training and testing process and cross-validation.

**Table 3:** Analysis of hyperparameter configurations for the methods employed.

Technique	Hyperparameter description
LR	max_iter=100, random_state=0, multi_class=’auto’, C=1.0
SVM	random_state=0,max_iter=500
GNB	var_smoothing=1e-9
RF	max_depth=100, n_estimators=100, random_state=0
GRU	loss = ’categorical_crossentropy’,optimizer = ’adam’,metrics=’accuracy’, activation=’softmax’, epochs=10
LSTM	loss = ’categorical_crossentropy’,optimizer = ’adam’,metrics=’accuracy’, activation=’softmax’, epochs=10

## 4 Results and discussions

This section presents a thorough evaluation of the models’ performance and efficacy in forecasting seizures based on EEG data. The results highlight the performance metrics of the applied AI models. The discussion delves into the significance of the obtained results, comparing them with existing literature and highlighting the novel insights achieved.

### 4.1 Setup for experiment

For experiments, we employed the Python programming language version 3.0 to build the artificial intelligence techniques utilized in this study. To conduct all experiments, we utilized the Google Colab open access environment [31], which provided a GPU backend along with 13 GB of RAM and 90 GB of disk space. To assess the effectiveness of the models, various performance metrics were utilized, encompassing accuracy, precision, recall, and F1 scores.

### 4.2 Results with original features

Table 4 presents the performance comparison results using the initial set of features. This evaluation assesses the performance metrics of both ML and DL learning models. Furthermore, it includes an examination of performance results categorized by class. The findings indicate that machine learning methods such as LR, SVM, and GNB exhibited suboptimal performance, with scores ranging from 0.22 to 0.44. Only the RF and GRU models achieved an average accuracy score of 0.69, although it is not the highest. This analysis concludes that there is still a need to improve the performance results of the applied approaches for the diagnosis of epileptic seizures.

**Table 4:** Performance comparative results analysis with original data features.

Model	Accuracy	Target class	Precision	Recall	F1 score
LR	0.22	epileptic seizure	0.25	0.39	0.30
		eyes closed	0.22	0.33	0.26
		eyes open	0.23	0.14	0.17
		healthy brain area	0.17	0.10	0.12
		tumor brain area	0.22	0.15	0.18
		Average	0.22	0.22	0.21
SVM	0.22	epileptic seizure	0.59	0.02	0.04
		eyes closed	0.26	0.31	0.29
		eyes open	0.22	0.27	0.24
		healthy brain area	0.20	0.33	0.25
		tumor brain area	0.19	0.17	0.18
		Average	0.29	0.22	0.20
GNB	0.44	epileptic seizure	0.94	0.82	0.88
		eyes closed	0.42	0.29	0.34
		eyes open	0.37	0.74	0.50
		healthy brain area	0.28	0.20	0.23
		tumor brain area	0.21	0.14	0.17
		Average	0.45	0.44	0.42
RF	0.69	epileptic seizure	0.93	0.96	0.94
		eyes closed	0.74	0.77	0.76
		eyes open	0.62	0.65	0.63
		healthy brain area	0.55	0.54	0.55
		tumor brain area	0.62	0.54	0.58
		Average	0.69	0.69	0.69
GRU	0.69	epileptic seizure	0.96	0.96	0.96
		eyes closed	0.53	0.59	0.56
		eyes open	0.44	0.45	0.44
		healthy brain area	0.85	0.72	0.78
		tumor brain area	0.71	0.73	0.72
		Average	0.70	0.69	0.69
LSTM	0.60	epileptic seizure	0.97	0.65	0.78
		eyes closed	0.48	0.23	0.31
		eyes open	0.48	0.58	0.53
		healthy brain area	0.56	0.74	0.64
		tumor brain area	0.60	0.78	0.68
		Average	0.62	0.60	0.59

### 4.3 Results with PCA features

For a fair comparison, we applied the PCA dimensionality reduction mechanism in this study, as described in Table 5. Using PCA, we selected 20 features from the EEG brain activity signals data. We calculated the performance results specifically for the best-performing method, RF, using the original features. However, this analysis shows that even with the application of PCA, poor performance scores were achieved. Therefore, additional scientific methods are needed to improve performance scores and effectively diagnose epileptic seizures.

**Table 5:** Performance comparative results with PCA features.

Model	Accuracy	Target class	Precision	Recall	F1 score
RF	0.66	epileptic seizure	0.92	0.97	0.94
		eyes closed	0.72	0.71	0.72
		eyes open	0.53	0.56	0.54
		healthy brain area	0.53	0.62	0.57
		tumor brain area	0.60	0.45	0.51
		Average	0.66	0.66	0.66

### 4.4 Results with transfer learning-based features

The results of applied techniques with new transfer features are given in Table 6. A new transfer feature set is generated using the proposed RFGR approach and used for building the learning approaches applied in this analysis. The results analysis shows that the applied techniques improve their performance scores with the proposed feature generation approach. The RF model achieves the highest performance score of 0.99, followed by the LR method. This analysis indicates that using a novel transfer technique for feature engineering has resulted in the highest performance scores for epileptic seizure detection in contrast to state-of-the-art methods.

**Table 6:** Performance comparative results with newly generated features.

Model	Accuracy	Target class	Precision	Recall	F1 scores
LR	0.98	epileptic seizure	1.00	1.00	1.00
		eyes closed	1.00	0.98	0.99
		eyes open	0.99	0.99	0.99
		healthy brain area	0.95	0.97	0.96
		tumor brain area	0.97	0.96	0.97
		Average	0.98	0.98	0.98
SVM	0.97	epileptic seizure	1.00	1.00	1.00
		eyes closed	1.00	0.99	0.99
		eyes open	0.96	0.99	0.97
		healthy brain area	0.96	0.96	0.96
		tumor brain area	0.97	0.94	0.95
		Average	0.98	0.98	0.98
GNB	0.97	epileptic seizure	1.00	1.00	1.00
		eyes closed	0.99	0.95	0.97
		eyes open	0.93	1.00	0.96
		healthy brain area	1.00	0.97	0.99
		tumor brain area	0.97	0.98	0.97
		Average	0.98	0.98	0.98
**RF**	**0.99**	epileptic seizure	1.00	1.00	1.00
		eyes closed	0.98	0.97	0.97
		eyes open	0.97	0.97	0.97
		healthy brain area	1.00	1.00	1.00
		tumor brain area	0.99	0.99	0.99
		**Average**	**0.99**	**0.99**	**0.99**
GRU	0.97	epileptic seizure	1.00	1.00	1.00
		eyes closed	0.95	0.98	0.96
		eyes open	0.97	0.94	0.96
		healthy brain area	1.00	0.98	0.99
		tumor brain area	0.97	0.98	0.98
		Average	0.98	0.98	0.98
LSTM	0.96	epileptic seizure	1.00	1.00	1.00
		eyes closed	0.87	0.99	0.93
		eyes open	0.99	0.86	0.92
		healthy brain area	1.00	0.95	0.97
		tumor brain area	0.94	0.98	0.96
		Average	0.96	0.96	0.96

**Fig. 4:**
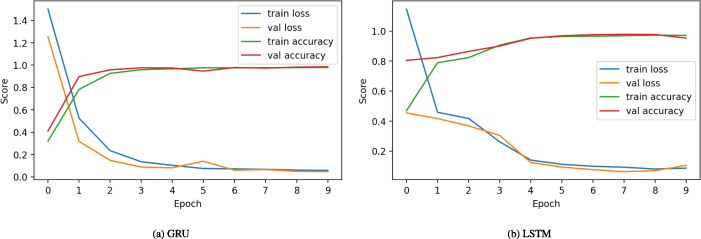
Analysis of deep learning methods during training with transfer features.

The figure illustrating the performance analysis of deep neural techniques applied to time series data can be seen in Fig. 4. The GRU and LSTM models are trained for 10 epochs. During the training process, the loss and accuracy scores are monitored. The examination indicates that during the first three training periods, there were elevated loss scores alongside diminished accuracy. Subsequently, starting from the fourth epoch, adjustments to model weights led to a progressive reduction in loss and improvement in accuracy throughout the remaining training epochs. The findings suggest that both deep learning techniques consistently achieved training accuracy exceeding 90 %.

**Fig. 5:**
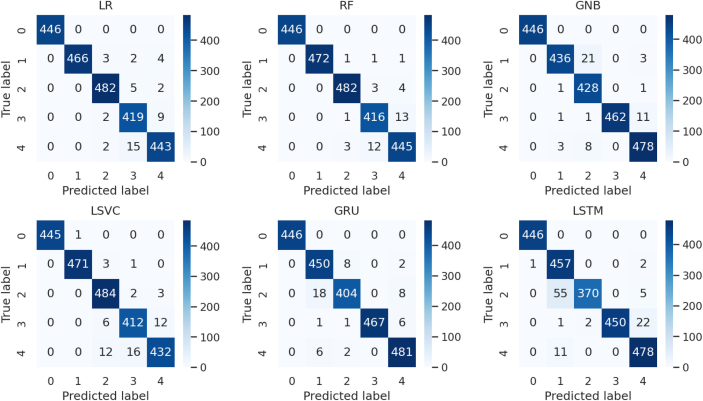
The confusion matrix analysis of the applied technique with newly generated features.

Fig. 5 presents the confusion matrix validation results of the implemented methodologies incorporating newly derived features. The results infer that the RF model accurately predicts brain activity target labels with minimal errors, contrasting with the LSTM approach, which exhibits higher errors and a resulting accuracy score of 0.96. This assessment confirms the effectiveness of all learning techniques utilized, leveraging the newly introduced feature engineering strategy.

### 4.5 K-fold validation results

The performance validation results of applied ML approaches are evaluated using k-fold validation, and results are given in Table 7. Both the original and transfer learning-based features are split into 10 folds to evaluate the generalization of each applied approach in this analysis. In this investigation, it is evident that utilizing original features yields subpar k-fold cross-validation outcomes. However, through the proposed method, we have successfully generated new transfer features that significantly improve k-fold cross-validation performance. Specifically, employing these new features, the RF model attained an accuracy score of 0.99 with a minimal standard deviation of 0.0021 in diagnosing epileptic seizures.

**Table 7:** Performance validation analysis based on k-fold cross-validation.

Technique	K-fold	With original features		With new transfer features	
		Accuracy	Std. Dev. (±)	Accuracy	Std. Dev. (±)
LR	10	0.22	0.0106	0.98	0.0037
SVM	10	0.21	0.0130	0.97	0.0089
GNB	10	0.43	0.0135	0.98	0.0036
**RF**	10	0.69	0.0117	**0.99**	**0.0021**
GRU	10	0.70	0.0173	0.97	0.0052
LSTM	10	0.65	0.0141	0.97	0.0056

### 4.6 Computational complexity results

The computational complexity analysis of applied learning methods is demonstrated in Table 8. The analysis is based on execution time (in seconds) using the original and newly generated transfer feature sets. The analysis shows that the original features are highly complex, resulting in high computation scores for the applied techniques in diagnosing epileptic seizures. The proposed feature engineering technique reduces the execution time substantially, with RF requiring only 1.727 s, which is the minimal time in contrast to the other models. This analysis demonstrates that the transfer features computation scores are lower than those using the original ones.

**Table 8:** The computational complexity analysis of applied models.

Technique	Runtime computations (seconds)	
	Original features	New transfer features
LR	0.957	0.185
SVM	14.46	0.095
GNB	0.033	0.008
RF	12.04	1.727
GRU	42.77	15.43
LSTM	25.13	16.86

Based on the results given in Table 8, the proposed model can achieve near real-time analysis, particularly when using optimized transfer features. However, the actual runtime may vary depending on hardware resources such as processing power, memory, and GPU capabilities. With adequate hardware, the reduced computational time achieved with the transfer features suggests that real-time analysis is feasible.

### 4.7 Results of ablation study

The ablation analysis conducted in Table 9 demonstrates the significant impact of the proposed features on model performance. When using the original data features, the RF model achieved an overall accuracy of 69.00 %, with class-wise F1 scores varying between 0.55 and 0.94. Notably, the model exhibited strong performance in detecting epileptic seizures (F1 score = 0.94) but struggled with tumor brain areas and healthy brain regions, where the F1 scores dropped to 0.58 and 0.55, respectively. In contrast, integrating the proposed features led to a remarkable improvement, elevating the model’s accuracy to 99.00 %. The refined feature set enhanced class-wise performance across all categories, achieving near-perfect precision, recall, and F1 scores. This stark performance boost highlights the effectiveness of the proposed feature engineering strategy in distinguishing different brain states, particularly in improving classification consistency for challenging categories such as tumor brain areas and healthy brain regions.

**Table 9:** Performance comparison of RF model with original and proposed features.

Feature Set	Accuracy	F1 score					
		Epileptic Seizure	Eyes Closed	Eyes Open	Healthy Brain Area	Tumor Brain Area	Average
Original Features	0.69	0.94	0.76	0.63	0.55	0.58	0.69
Proposed Features	0.99	1.00	0.97	0.97	1.00	0.99	0.99

### 4.8 Comparison analysis of feature space

**Fig. 6:**
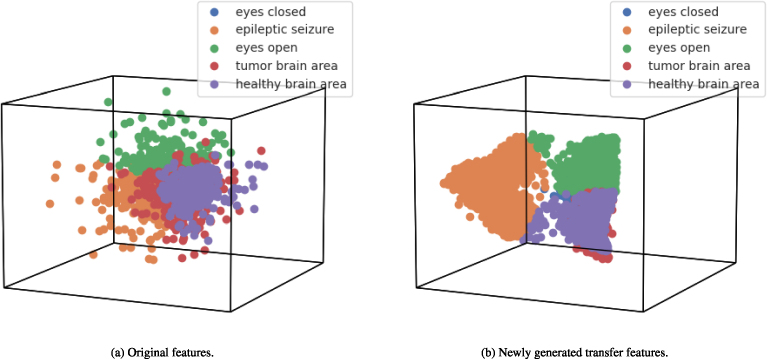
The feature space results are based on each class distribution.

The feature space comparison analysis in a 3-dimensional space is depicted in Fig. 6. The feature space analysis demonstrates that the original EEG signal data is highly complex and not easily separable by the applied learning techniques. This complexity is the primary reason for the lowest performance scores obtained with the original dataset features. However, the newly generated transfer features, obtained through the proposed feature engineering approach, exhibit a high degree of separability. This enhanced separability of the dataset contributes to the validation of high-performance scores for diagnosing epileptic seizures in this study.

### 4.9 Analysis using explainable AI

**Fig. 7:**
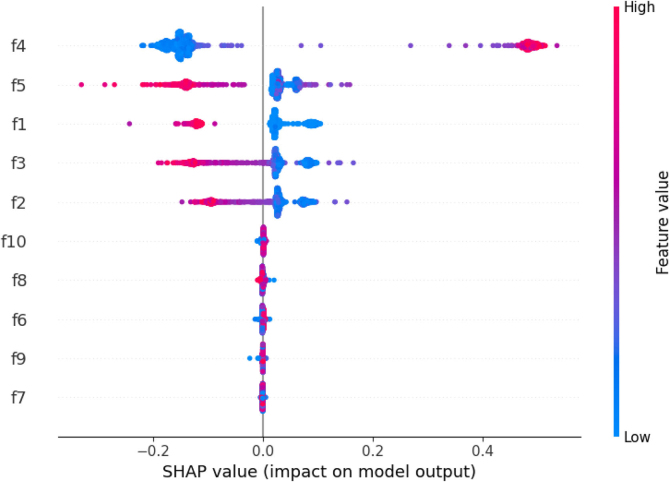
The proposed approach utilizes SHAP charts and random forest (RF) analysis to make decisions regarding the diagnosis of epileptic seizures.

The SHAP method is employed to identify the features of brain activity signals based on their contribution to the predictions made by the proposed model, as depicted in Fig. 7. The SHAP results consist of a list of transfer features with importance scores arranged in descending order. The analysis demonstrates that the transfer features f4, f5, f1, f3, and f2 play a significant role in diagnosing epileptic seizures.

**Fig. 8:**
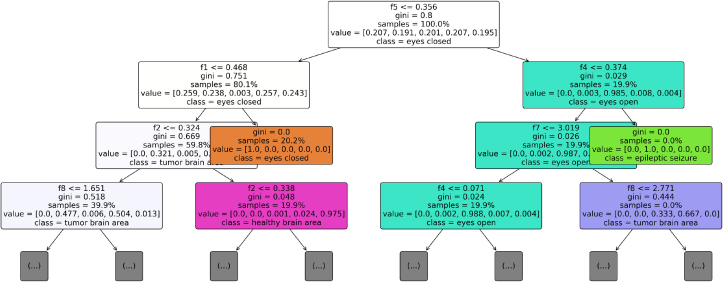
TreeMap-based proposed RF technique decisions marking analysis for diagnosis of epileptic seizures.

Subsequently, the TreeMap approach is utilized to gain valuable insights into the behavior of the RF model, as shown in Fig. 8. The analysis allows us to understand how the proposed model reaches its conclusions regarding the diagnosis of epileptic seizures in patients.

### 4.10 State-of-the-art methods comparison

Table 10 presents a comparison of performance with recent studies in the field. Previous studies employing the identical dataset were considered for this evaluation. Results show that accuracy scores ranged from a minimum of 80 %.

**Table 10:** Comparison of performance with existing studies utilizing the same dataset.

Ref.	Learning type	Proposed technique	Accuracy
[19]	Machine learning	Support Vector Machine	97.00 %
[32]	Deep learning	Artificial Neural network	80.00 %
[33]	Deep learning	SOM-RBF	97.47 %
[34]	Machine learning	SVM and RF	93.10 %
[35]	Deep learning	Artificial Neural network	97.55 %
**Proposed**	**Transfer learning**	**Novel RFGR + RF**	**99.00 %**

### 4.11 Validation using external dataset

The proposed model was further validated using an external dataset [36] of patient ECG readings, and the results are summarized in Table 11. The model achieved an overall classification accuracy of 98 %. For the normal class, the model attained a precision of 0.98, a recall of 0.99, and an F1-score of 0.98, indicating highly reliable detection of normal cardiac signals with minimal false negatives. Similarly, for the abnormal class, the precision, recall, and F1-score were consistently high at 0.99, reflecting the model’s robustness in identifying pathological patterns without significant loss of sensitivity or specificity.

**Table 11:** Proposed model results with external data of ECG readings of patients.

Accuracy	Label	Precision	Recall	F1
0.98	normal	0.98	0.99	0.98
	abnormal	0.99	0.99	0.99

### 4.12 Discussions

Through a comprehensive analysis, it is concluded that the incorporation of hybrid features significantly contributes to achieving better results in disease diagnosis using ECG data. The integration of temporal features extracted by a gated recurrent unit and probability-based features extracted by RF in the RFGR approach proves to be a pivotal factor in its effectiveness for diagnosing diseases based on ECG data. Overall, the proposed approach has made significant advancements in the early diagnosis of epileptic seizure disease, thereby contributing to improved patient care and the overall advancement of medical science.

### 4.13 Practical implications

This research has practical implications both for clinicians and researchers. For clinicians, the proposed approach provides a potential tool for the early and accurate diagnosis of epileptic seizures, which can lead to timely interventions, improved patient outcomes, and optimized treatment plans. The use of EEG signal data allows for non-invasive monitoring of brain activity, making it accessible and practical in clinical settings. Additionally, the integration of ML models in detecting seizures can assist healthcare professionals in reducing the likelihood of misdiagnosis, which is crucial for effective patient care.

For researchers, this study offers a novel ML-based approach that can serve as a foundation for further exploration in the field of neurological disorders. The methodology and findings can be leveraged to enhance existing models, develop advanced techniques for signal processing, and explore applications in related neurological studies. Furthermore, the dataset and feature extraction techniques used can be extended to other cognitive and neural disorders, fostering interdisciplinary research and innovation in biomedical engineering and neuroscience.

Overall, this research aims to bridge the gap between computational neuroscience and clinical applications, promoting evidence-based decision-making in the diagnosis and management of epileptic seizures.

### 4.14 Web-based graphical user interface

**Fig. 9:**
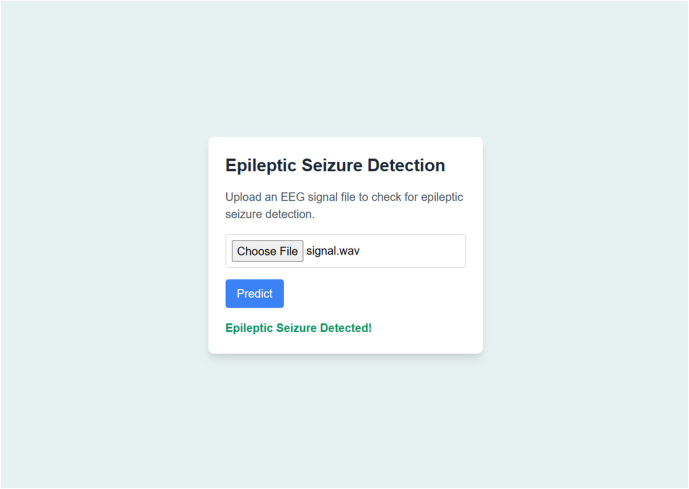
A web-based graphical user interface (GUI) that empowers real-time monitoring of epileptic seizure patients in hospital settings.

We designed a web page that serves as a prototype tool for the early diagnosis of epileptic seizures using EEG signal data, as shown in Fig. 9. The interface allows users to upload an EEG signal file in.wav format. Upon clicking the “Predict" button, a simulation of the ML model’s output is displayed, indicating whether an epileptic seizure is detected. In a practical implementation, the uploaded EEG signal would be processed through a trained proposed model to analyze patterns and predict the presence of epileptic seizures. This approach aims to assist healthcare professionals by providing a rapid and accessible preliminary assessment, aiding in the timely diagnosis and management of epilepsy. This prototype web-based GUI specifies the test environment (Windows platform with 12 GB RAM and a supported graphics card). The code for the web-based GUI is available at https://github.com/alirazadeveloper/Epileptic-Seizures-Detection-App.

## 5 Conclusions and future works

This research aims to develop an effective approach for the early prognosis of epileptic seizure disease using electroencephalography (EEG) brain activity signals. We introduced a novel transfer technique called RFGR for feature engineering, which involved the utilization of machine learning and deep learning approaches. The proposed feature generation approach generates a new feature set that proves to be more effective for training models. For performance evaluation, several experiments are carried out using the original feature set, the principal component analysis (PCA)-based feature set, and the feature set from the proposed approach. The experimental findings indicate that employing the suggested method enhances model performance significantly, with random forest (RF) achieving the highest accuracy of 99.00 %. In addition, the computational complexity of the proposed approach is also low, which demonstrates its practicality and efficiency in real-world applications. For further validation, k-fold validation is also utilized, which confirms the superior performance using RFGR-based features. Furthermore, performance analysis with existing state-of-the-art approaches reveals that the proposed methodology outperforms them. Additionally, we incorporated explainable artificial intelligence analysis techniques, such as SHAP charts and model decision trees, to provide transparent and understandable explanations of the decision-making processes employed by the proposed techniques in diagnosing epileptic seizures.

## CRediT authorship contribution statement

**Erol Kına:** Writing – original draft, Data curation, Conceptualization. **Ali Raza:** Writing – original draft, Formal analysis, Conceptualization. **Prudhvi Chowdary Are:** Methodology, Formal analysis, Data curation. **Carmen Lili Rodriguez Velasco:** Methodology, Investigation, Funding acquisition. **Julien Brito Ballester:** Visualization, Software, Project administration. **Isabel de la Torre Diez:** Visualization, Project administration, Investigation. **Naveed Anwer Butt:** Validation, Software, Formal analysis. **Imran Ashraf:** Writing – review & editing, Validation, Supervision.

## Funding

This research was supported by the European University of the Atlantic.

## Declaration of competing interest

The authors declare that they have no known competing financial interests or personal relationships that could have appeared to influence the work reported in this paper.

## Data Availability

The dataset used in this study is available at the following link: https://www.kaggle.com/datasets/harunshimanto/epileptic-seizure-recognition
